# Health workers’ social networks and their influence in the adoption of strategies to address the stillbirth burden at a subnational level health system in Uganda

**DOI:** 10.1371/journal.pgph.0000798

**Published:** 2022-07-25

**Authors:** Eric Ssegujja, Isaac Ddumba, Michelle Andipatin

**Affiliations:** 1 Department of Health Policy Planning and Management, School of Public Health, College of Health Sciences, Makerere University, Kampala, Uganda; 2 Faculty of Community and Health Sciences, School of Public Health, University of the Western Cape, Cape Town, Republic of South Africa; 3 Department of Health, Mukono District Local Government, Mukono, Uganda; 4 Faculty of Community and Health Sciences, Department of Psychology, University of the Western Cape, Cape Town, Republic of South Africa; The Hospital for Sick Children, CANADA

## Abstract

Health workers’ peer networks are known to influence members’ behaviours and practices while translating policies into service delivery. However, little remains known about the extent to which this remains true within interventions aimed at addressing the stillbirth burden in low-resource settings like Uganda. The objective of this study was to examine the health workers’ social networks and their influence on the adoption of strategies to address the stillbirth burden at a subnational level health system in Uganda. A qualitative exploratory design was adopted on a purposively selected sample of 16 key informants. The study was conducted in Mukono district among sub-national health systems, managers, health facility in-charges, and frontline health workers. Data was collected using semi-structured interview guides in a face-to-face interview with respondents. The analysis adopted a thematic approach utilising Atlas. ti software for data management. Participants acknowledged that workplace social networks were influential during the implementation of policies to address stillbirth. The influence exerted was in form of linkage with other services, caution, and advice regarding strict adherence to policy recommendations perhaps reflective of the level of trust in providers’ ability to adhere to policy provisions. At the district health management level and among non-state actors, support in perceived areas of weak performance in policy implementation was observed. In addition, timely initiation of contact and subsequent referral was another aspect where health workers exerted influence while translating policies to address the stillbirth burden. While the level of support from among network peers was observed to influence health workers’ adoption and implementation of strategies to address the stillbirth burden, different mechanisms triggered subsequent response and level of adherence to recommended policy aspects. Drawing from the elicited responses, we infer that health workers’ social networks influence the direction and extent of success in policy implementation to address the stillbirth burden at the subnational level.

## Introduction

Subnational health systems are formal structural collaborations of health workers and other health service providers which aim for a stronger service delivery structure [1–3]. The intertwined web of relationships brings together public facilities, private providers, health systems managers, professional associations, civil society, political leaders, and referral systems actors. Health workers interact regularly and rely on their relationships among peers to share patients, effect referrals, policy, and professional advice while attending to service delivery bottlenecks [4,5]. Physician peer networks are essential in influencing individual health worker behaviours [6,7]. Belonging to a network may determine the pace of adoption of new policies through social influence; a process by which network members are impacted by the medical practices of their peers through imitation, role modelling, and persuasion [1,8,9].

Social contagion theory postulates that social networks have a greater impact on provider-related behaviours and practices among health workers and their peers [6,10]. Globally, health workers turn to peers as reference points for policy advice and the adoption of new guidelines [11]. Peer influence in provider practices and patterns is reported to be pronounced in healthcare and policy implementation settings [5,6,11,14]. The peer’s adoption of new treatment technology led to the subsequent adoption of the same in a different setting [12]. Further, uptake of medical practice was noted to have been influenced by network connections among physicians [3,13]. By sharing patients, adoption of policy recommendations is likely accelerated following social contagion either because they are directly influenced or are in a similar position within the network [9,14]. Health workers often call upon colleagues for clinical advice, effect referrals, as administrative requirements, or second-opinion policy guidelines backstopping which are key sources of evidence on complexities surrounding policy translation at the final point of service delivery [5,10,11].

In Uganda, reasons for variations in policy implementation with a potential for health workers’ social networks to influence their direction exists. Lack of framework for systematic dissemination of guidelines, guidelines not being readily available at the service delivery level together with the adoption of a broader definition of target audiences for policies may compel health workers to turn to their workplace networks for support and guidance regarding the implementation of policies [15]. Further, inadequate support supervision, together with practices of lower cadres performing duties of specialists may too catalyse workplace peers to step in to guide policy implementation to bridge this gap [16]. Mukuru et al highlighted contextual factors, inadequate supplies, inputs workforce size, and skills mix compelled frontline workers to devise ways of ensuring continuity of health service provision which did not necessarily align well available policy provisions [17]. Contextually, little remains known about the influence of social networks in the adoption of strategies to mitigate the stillbirth burden in Uganda. At the subnational level, health worker social network relationships can support policy implementation through enforcement of procedural aspects such as support supervision, referral patterns, and sharing of clinical advice. When explored, these patterns can unveil channels through which policy diffusion informally occurs between providers. Understanding the nature of such a relationship is key in generating evidence on how local practice patterns support interventions to address stillbirth at the subnational level.

Translation of policies to address the stillbirth burden provides a good case study. For international comparisons, stillbirth refers to fetal loss after 28 weeks of gestation and it is the same used in Uganda [18]. Following the global campaigns [19], Uganda took drastic measures to translate Every New-born Action Plan (ENAP) targets into the Reproductive Maternal New-born Child and Adolescent Health (RMNCAH) Investment case which is the flagship national Maternal and Child Health (MCH) umbrella policy guideline. Subsequent efforts saw the translation of these at the subnational level into actual service delivery. At the time (2013/5), national stillbirth rates stood at 21/1000. The ENAP target for countries aimed to reduce the rate by at least 12/1000 or less by 2030. The current trend reflects the improved performance of this particular indicator which stands at 9/1000 facility-level live births [20,21]. Despite this positive trajectory, emerging evidence also suggests that most stillbirths are currently occurring from within health facilities. This reflects the poor quality of MCH services delivered and the important role that subnational health systems can play in addressing the emerging pattern of stillbirths happening within health facilities. With the 2030 target around the corner, a need for acceleration of efforts to address possible bottlenecks will be key.

Moreover, the available evidence in response to this phenomenon is predominantly from high-income countries where health systems are perceived to be more functional and with the lowest burden of stillbirth. In this study, we considered the relationship to exist when health workers shared the provision of maternal healthcare to mothers and exchanged policy or professional advice in efforts to mitigate the stillbirth burden. The objective of this study was to explore experiences and processes through which health workers’ social networks contributed to strategies to address the stillbirth burden at the subnational level in Uganda. To our knowledge, this is the first to document the influence of health worker social networks in addressing stillbirth thus contributing to the current evidence regarding factors that underly translation of stillbirth reduction strategies at the subnational level.

## Materials and methods

### Study design

The study adopted a qualitative exploratory design. We aimed to understand the influence of health worker social networks in addressing the stillbirth burden. Subnational health systems adopted policy priorities highlighted at the national level in a bid to address stillbirth in Uganda after global campaigns guided national level efforts which gained momentum after 2011. Additional material regarding the applied methodology has been published elsewhere [21,22].

### Setting

The national health sector aspirations are reflected in the national health policy II and the five-year health sector strategic and Investment plan (HSSIP 2021–2026). The national health system is organized around a tiered health structure whereby at the top is the Ministry of Health (MoH) followed by the national referral hospitals, regional referral hospitals, district health management teams, district hospitals, Health Centre four (HCIV), Health Centre Three (HCIII), Health Centre Two (HCII) which is the first level of clinical care and the Village Health Teams (VHT) structure that operates as the community arm of the health system. They are interlinked through a detailed referral system which has been enhanced through the recent introduction of the referral policy and national ambulatory policy.

The country operates a Pluralistic health system with multiple actors including the public sector and the private providers. Among the private providers are private-not-for-profit (PNFP) under the different religious medical bureaus and the private-for-profit (PFP) that include high-level corporate hospitals and lower-level clinics. These are guided through the Public-private partnership policy for health (PPPPH). Overall, the public sector constitutes 55% of care facilities, 29%, and 16% by the PFP and PNFP respectively. At the macro level, the MoH leads the stewardship taking on the policy formulation functions in addition to planning, coordination, quality assurance, and resource mobilization. It is at this level that global stillbirth campaigns were translated into national policy objectives and reflected in the different MCH policy documents. Under the decentralization for health arrangement adopted in 1997 as part of the comprehensive decentralized system in Uganda, decentralization responsibilities are vested at the subnational level with the district political leadership Local Council Five (LCV) responsible for providing oversight to the health service delivery in the district. Unique to health services, a health sub-district level was introduced.

Maternal and child health services at the subnational level is delivered through the routine standard of care under the direct supervision of the District Health Office (DHO) where the Assistant DHO, a post usually designated for a senior nurse act as the focal person for MCH in the district. Following efforts for interventions to achieve Millennium Development Goals (MDG), particularly those related to goals 4 &5, the health sub-district was staffed with a physician as the in-charge to operationalize the Comprehensive Emergency Obstetric and Neonatal Intensive Care (CEmONIC). Following Every New-born Action Plan (ENAP) recommendation [20], targets were translated into national policies where strategies to address stillbirth are implemented at all levels of service provision. Currently, the stillbirth rate is one of the indicators considered for assessing the annual district performance and a quality-of-care indicator at the health facility level.

It is within this context that this study was conducted in a peri-urban subnational level district that was at the time of the study considered among districts with a high stillbirth burden.

### Population and sample

Details about the study participants are described elsewhere [22]. The process involved purposively identifying six health facilities and the district health management team from where potential respondents were identified. The six health facilities included two hospitals, two Health centre IVs, and two Health centre IIIs. By ownership, the two hospitals were private-not-for-profit facilities affiliated to the catholic medical bureau and protestant medical bureau respectively while the remaining four were public health facilities. Sixteen respondents were purposively selected and included in the final sample upon which no new data insights were emerging and therefore presumed to have attained data saturation at that point. Some respondents were occupying more than one position of responsibility which was a target for our interviews. The sampling strategy involved targeting health facility in-charges, maternity unity, and antenatal care unit in-charges, health workers specifically nurses, midwives, and doctors working in those units. At the district and sub-district level, the strategy involved purposively targeting the health managers directly involved in the delivery and supervision of maternal and child health services within their areas of jurisdiction. Overall, all had spent two or more years in their current positions. For example, some midwives acted as maternity unit in-charges while the subdistrict head also doubled as heath facility in-charges. Overall, none of the potential respondents identified and approached declined to participate.

### Data collection and analysis

After introducing the study to the district health office, consultations were made about potential respondents. This led to a compilation of a list of people with contacts to be approached later by the study team. The same process was repeated at each of the health facilities that were visited during the study period. Potential respondents were then approached with the purpose of the study explained. For those that expressed willingness to participate, a date and secure venue for the interview were agreed upon. Overall, none of the contacted potential respondents declined to participate. On the day of the interview, written informed consent to participate in the study was obtained before the actual face-to-face interviews were conducted with a study team member. Data collection happened at the respondent’s places of work and was conducted by the first author (male) with a strong grounding in qualitative research methods, and working experience of health systems strengthening particularly maternal and child health interventions. He was supported in data collection tasks by a female graduate-level research assistant with experience in qualitative data collection and familiar with sub-national health systems and maternal and child health service delivery. Both conducted interviews separately with the criteria for determining who conducted which interview not restricted to the interviewer’s gender. All interviews were conducted using semi-structured interview guides in a face-to-face interview with respondents. Data capture was by use of notebooks, field notes, and digital audio recorders. At the end of each field day, the audio files were downloaded and saved on a password-protected laptop computer, and a copy was saved on an external hard drive. The field notes which were taken during the interviews were expanded to cover relevant insights to the study objectives which were observed but could not be captured with the audio recording. On average, the interviews lasted between forty-five minutes to one hour. Apart from the respondent and interviewer, no other persons were present during these interviews. Interview transcriptions were done verbatim using Microsoft office word since all interviews were conducted in English. We did not return the transcripts to respondents for comments after the exercise. The questionnaire used was developed for this study and informed by the literature. It was piloted before data collection and has been previously published elsewhere [22]. Briefly, it had eight questions that explored the health worker’s experiences, and the role of both facility management and individual health workers in ensuring that stillbirth risk factors were responded to appropriately. It further explored the influence of the workplace context and mothers’ characteristics in translating guidelines at the frontline.

Analysis followed a thematic technique. After a verbatim transcription process which saw typing out all audio interviews into micro soft office word and uploaded into Atlas. ti, a qualitative data management software together with the expanded field notes. Deductive data coding was done by the first author after a rigorous exercise of developing the final codebook that was used. The process involved first; an inductive coding on a sample of five transcripts to identify emerging themes which were later used as codes that formed the codebook. These were directly related to the study’s main objective of the influence of social networks in the adoption of strategies to address stillbirth. The coding exercise involved assigning all relevant textual data to a particular code. Query reports were run for each of the codes and a manual pile sorting process involved grouping text with similar meaning into the same piles by the first author (ES). These were further analysed inductively for emerging sub-themes. Triangulation involved a comparison of results from the audio transcripts with field notes as well as a contrast of textual data from the different categories of respondents. Feedback on the contextual interpretation of results was provided by the second author (ID) while input on the codebook development, coding process, theme development, and triangulation of results was provided by the third author (MA). The findings are what have been used in the presentation and interpretation of results with representative quotes used in some instances to bring out the participants’ voices. Finally, we followed the Consolidated Criteria for Reporting Qualitative Research (COREQ) while reporting the qualitative results (S1 COREQ Checklist).

#### Ethics approval and consent to participate

The entire study was performed in accordance with the Declaration of Helsinki for which ethical approval was obtained from the University of the Western Cape Biomedical Research Ethics Committee (BM/17/9/1). In Uganda, the study was reviewed by the Makerere University School of Social Sciences Research and Ethics Committee (MAKSS REC 12.17.110) and the Uganda National Council for Sciences and Technology (SS 4575). Permission to conduct the study as part of the administrative clearance was obtained from the district authorities and health facility administration to access the respondents. Informed consent was secured from all respondents before interviews commenced.

## Results

### Health facility workplace social networks

**Fig 1:**
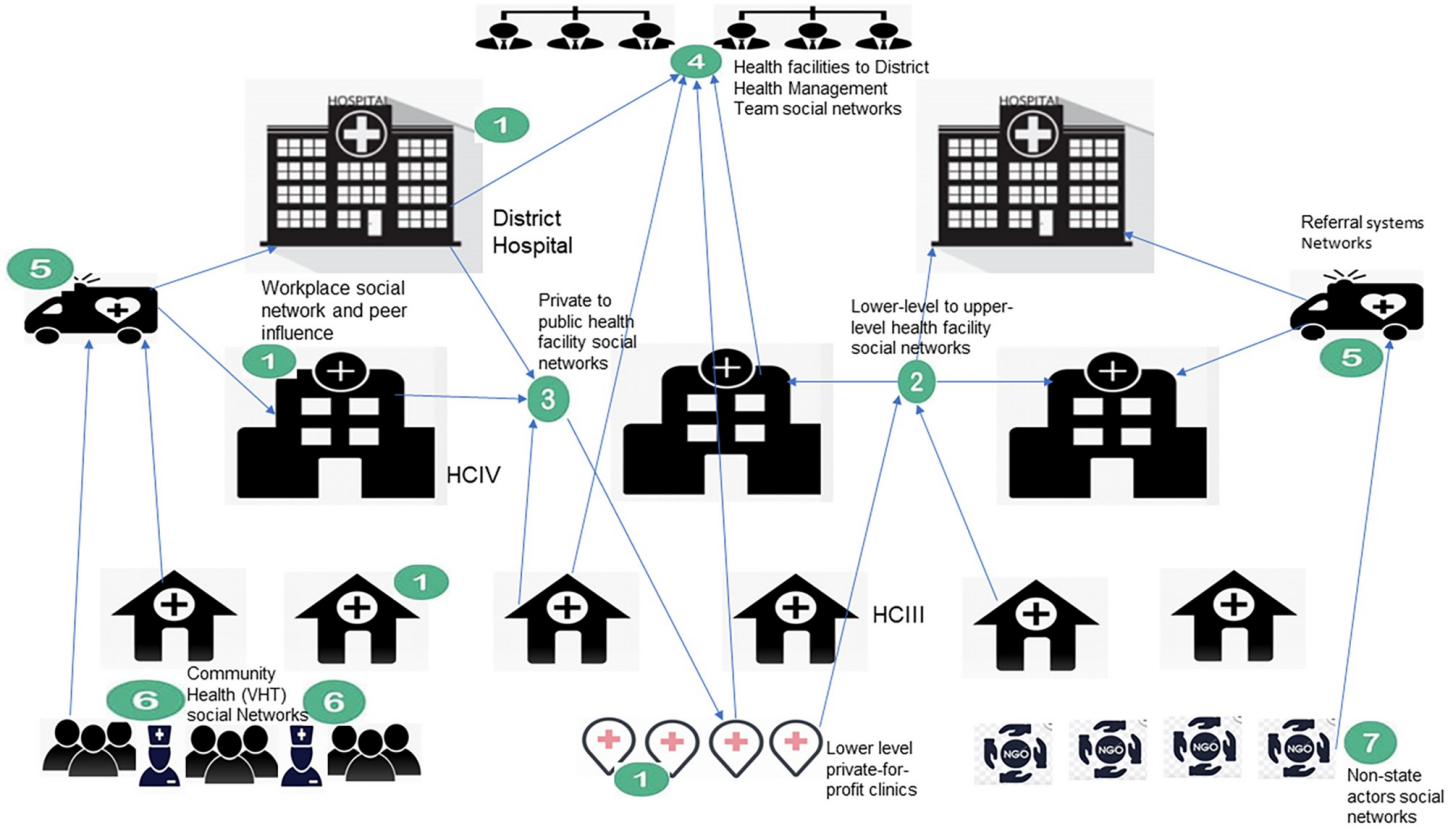
Pictorial flowchart of social network relations.

Relationships among health workers within the same physical workplace formed the most pronounced level where network members interacted regularly to improve the delivery of services to address stillbirth risk. Fig 1 provides a pictorial illustration of these relationships and interactions. It involved both formal and informal engagements through which network members influenced the adoption of strategies to address stillbirth. The mechanisms of influence included; coaching which embraced “learning by doing”, expert consultations from senior colleagues, occasional reminders about guidelines enforcement, and peer learning through organized sessions during CMEs as reflected in the quotation below;

*we go and look for those particular sessions that maybe need urgent attention*. *So*, *through CMES are general to everyone sometimes we ensure that CMEs are particular to the midwives so that we can maximize their attention*.

***PNFP*_*Hosp*_*001*_*IC*_*Mat***

Other formal mechanisms involved the implementation of quality improvement assurance measures, skills-building to address identified gaps, support supervision, and the different interfaces through which staff feedback meetings were held as reflected in the quote below;

*during the meetings*, *we discuss some of those things and in case you are managing a condition and you need to consult on something you can refer to the guidelines and come back and manage the cases*.

***PF*_*HCIII*_*005*_*IC*_*Mat***

Instances of negative influence from workplace social networks while implementing intervention to address stillbirth burden were reported. It emerged that cases of slack adherence to guidelines were not an exception which could have resulted in the compromised quality of maternal and child health services delivered. A common practice reported involved health workers shielding their absentee peers from disciplinary and recourse procedures other than subjecting them to the same which could have affected the delivery of health services. Cases of available health workers covering up their absentee peers to deliver services including particular tasks not designated for their cadreship, especially during night shifts were reported. Relatedly, some workplace relationships extended into such levels where the supervisor’s objectivity in enforcing strict adherence to guidelines were compromised. In such cases, provision of supervision feedback to colleagues perceived as close friends became such a challenge. In one of the interviews, it was observed that;

*You know this bit of friendship where I feel you are my colleague and if there was a mistake and its attributed to you such as delaying to [report for work]*, *somehow I fail to caution you about [the exact mistake]*.

***PF*_*HCIV*_*004*_*MW***

### Lower-level to upper-level health facilities networks

Health workers’ social networks were reported to support the identification and recommendation for appropriate maternal healthcare according to mothers’ conditions which informed the referral decision. These ranged from requirements for fetal evacuation, C-section, and blood transfusion services among others. Existing relationships from network members were used to initiate advance contact before effecting referrals to save time, especially in response to addressing the second and third delays while managing mothers as reflected in the quotation below;

*We know some of the facilities around and the staff working there* … *Sometimes the midwife can tell you that this mother was referred at such and such a time and we can compare the time of referral and time of arrival at the facility*. *If I fail on the phone sometimes*, *we meet and discuss*, *that you know what you sent us a mother with such and such a condition and the outcome was this*. *So if it was her negligence she changes if she has a positive attitude*.

***PF*_*HCIV*_*004*_*IC*_*Mat***

Health workers’ social networks with workers at other facilities were part of more enduring relationships and were missing in underserved areas. In settings where they existed, they supported in re-assessment of referred patients to identify inherent skills gaps at the referring entities which were unveiled and addressed to prevent future re-occurrence. While supporting network members, it emerged that such support was not a one-off engagement but rather embedded in long-term relationships which was not the case when cases from health facilities far away from/outside the district were being managed. For facilities directly under their oversight, network members engaged in support supervision and review of MCH activities in general that lower-level facility health workers provided for compliance with policy guidelines and technical backstopping. In underserved areas that lacked such closely-knit networks, especially in the hard-to-reach areas like the islands, these benefits were reported to be missing as reflected in the quotation below;

*we have a strong workforce; we have two medical officers who have tried to do a lot of supportive supervision for the lower-level health facilities*. *[but] because [our] health subdistrict comprises some island*, *what happens there nobody knows much as we have government health facilities there but we cannot be sure what goes on there in time*.

***PF*_*HCIV*_*003*_*MO***

### Private to public health facilities’ social networks

Health worker social networks extending into the lower-level private-for-profit maternity care centres were engaged by health workers from public health facilities that had a direct supervision mandate. They provided advice to health workers at lower-level private-for-profit maternity care centres in form of caution on service provision capacity according to the level of operation. This enabled health workers at private maternity centres to identify mothers whom they were not mandated to manage at their level of service provision and initiate early referrals or provide advice to them on where they could easily access the required service. Among lower-level private maternity centres where monitoring was not very strictly adhered to, health workers from the supervising entities (public health facilities) were reported to offer advice and mentorship on the appropriate use of drugs to prevent misuse of oxytocin during labor. The same networks were used by health workers from public health facilities to support data capture at the private maternity care centres. In addition, the adoption of proven evidence-based practices like the use of partograph for labor monitoring was encouraged among private maternity care centres. Such capacity-building efforts through mentorships reflected initiatives for establishing and maintenance of a long-term relationship aimed at improving the quality of MCH services at the subnational level.

*So*, *they usually come with the nurse and we usually first work with her*. *Like you show her what the problem is which she should have worked on before the referral and when all that is done that is when the nurse leaves*. *For all the private clinics that have initiated referrals that is how it is done*. *Initially*, *they would come and brought a patient for you and would go*. *But we have devised a means to try and mentor them*. *So*, *we wouldn*’*t allow them to go without knowing what has been done and what the outcome is*.

***PNFP*_*Hosp*_*002*_*MO***

Other efforts included looping lower-level private maternity service providers into the subnational referral system by providing them with referral forms and telephone contacts on which to call while initiating referrals. Same relationships were used to receive feedback about patient management as echoed by a respondent in the quotation below;

*what we have been emphasizing is these private practitioners who are referring to us are getting referral forms from the sites where they are referring these mothers*. *Good enough sometimes they even escort them to the hospital*. *For me when I get any patient without a documented referral*, *I make sure if they don*’*t have those forms in their health facilities*, *I make sure they pick those forms and I encourage the referring health worker to write to us about what was given to this mother*.

***PNFP*_*Hosp*_*001*_*IC*_*Mat***

Instances detrimental to the network aspirations were also registered. A case in point was when private providers preferred to refer cases outside the district other than first seeking referrals from within the subnational health systems. This was particularly true when similar services were offered for free at public health facilities or costed a relatively small fee at PNFP but mothers would instead be referred to public facilities located particularly in the capital city which was a relative distance outside the district. Other instances included private-for-profit actors holding onto mothers for far too long over cases they could not manage and were aware of where such services could be offered in other health facilities within the districts. The main reason to have driven this behaviour was reported to be a dislike for competition. It was suspected that within district referrals would portray a particular private maternity centre as incompetent and lacking in capacity to provide care or manage certain maternal health conditions before their potential service users. A feeling that once mothers were referred to nearby alternative maternal health service providers would not come back to receive services in future from the referring facility was evident. The same feeling was reported to be the underlying motivation for the undesirable practice of private maternity service providers holding on to parturient mothers with conditions beyond their capacity to manage for far too long hence exposing them to further risk. A respondent observed;

*you know for them [private maternity centres] they feel like they should handle until the last minute but when they fail at times they go to [health facility located in the capital city which is outside the district]*. *They feel like they should treat them until they feel they cannot handle it anymore*. *So the mothers are getting complications from those facilities*.

***PF*_*HCIV*_*003*_*MW***

### Health facility to district health management networks

District health managers formed the apex of the subnational health system decision-making body. They superintended all actors contributing to the improvement of the quality of maternal healthcare. In that role, they emerged as key actors within these social networks where they received surveillance notification for all perinatal death from health facilities which information, they would use to seek an explanation of the cause then tailor Continuous Medical Education (CME) sessions to address the cause and request that perinatal death review (PDR) was conducted. In case of high rates, they would inform health facility level maternal service providers of the district’s concerns about the emerging trends originating from their respective health facilities. Engagement with particular health workers who in turn shared these concerns with peers at the workplace reflected typical scenarios of performance improvement utilizing the existing network relationships. District quarterly support supervision visits were conducted through which contextual bottlenecks were identified and strategies to address them devised. The Medical superintendents also performed health subdistrict management roles which mandated them to ensure quality MCH service delivery at both the health facility and subdistrict level. Regular meetings convened by the DHO were organized with participants including maternal health service providers. These acted as platforms from which network actors engaged and exchanged views on top of reviewing submitted data to analyze emerging trends. They were the main platform where stakeholders would interact and share implementation experiences and advice. The quotation below puts it in perspective;

*at the district level*, *we mainly address this issue when we look at the data that comes from these health facilities*. *Obviously*, *our first question is why*? *In most cases*, *we call the specific health facilities*. *To begin with*, *you start by confirming the data*. *Then you talk to the in-charge and they give you most of the reasons which in most cases we are all familiar with; like these have been referrals-in which came in very late having spent time at the private clinics and things like that*.

***DHMT*_*007*_*Nur*_*02***

### Referral systems networks

A unique network of actors converging around the subnational level referral system was established. This comprised of individual health workers, ambulance drivers, Non-Governmental Organisation (NGO) actors, health facility in-charges, and Health system managers both at the district and health sub-district levels. These network members played varying roles in ensuring the functionality of the referral system. At the individual level, health workers provided their telephone contacts to pregnant mothers which they could call for consultations in case of quick maternal health advice rather than going to the health facility to seek the same. These efforts were boosted by an NGO to all facilities providing Comprehensive Emergency Obstetric Care services (CEmONIC) with a toll-free line from where mothers would call directly to the health facilities free of charge. Private ambulances were provided by local area politicians like Members of Parliament (MP) under a cost-sharing arrangement where fuel costs would be met by the users. These supplemented both Government and Private-not-for-profit (PNFP) referral vehicles with toll-free hotlines and networked lower-level health facilities that were encouraged to call in cases where they required pick-ups from their health facilities. Additionally, the public facilities linked to the national ambulatory system from where their in-charges can contact and request transportation in case they want to refer a mother to a tertiary level facility outside the district for further management of complicated cases.

*we would call the in-charge to come and then if the [mother required] a referral*, *[the in-charge would] be the one to provide transport and we refer the [mother] to the hospital*. *It’s not that we have a facility ambulance but some referrals may be because a mother may present with a previous scar while she was [attending] ANC and you told her to deliver from where they [could] do a c-section*. *We [advise] them to either go to a hospital or HCIV but sometimes you realize that such [mothers would] again present here [while] in labour and there is no way [we] chase [them] away so [we just] counsel and refer [them] to the hospital again*.

***PF*_*HCIII*_*006*_*IC*_*Mat***

A practice was observed where the referred mothers would decline advise given and instead opt to go to the nearby health facilities for services even when the service needed was unavailable. Sometimes they were reported to even end up delivering from the Traditional Birth Attendants (TBAs). The underlying driver being the fear that the referral process would make them incur more financial and time expenses that they did not have at the time or intend to partake in the process.

### From community to health facilities social networks

In the community, Village Health Teams (VHT) with support from partners were engaged in community-level maternal health promotion activities like the distribution of mama kits to pregnant mothers in effort to promote facility deliveries. There were observed cases where community-level actors within these networks supported improvements in the documentation of shared patients. Specifically, a case was reported of a private midwife offering maternity services maintaining a strict documentation routine that public health facility nurses had to comply with as reflected in the quote;

*there is a private midwife who has a maternity home*. *For her she is strict with her maternity center*… *when they are referring to us there is a book and a referral note where they write and you document as you receive this patient and when you receive*, *they even put your comments*. *Like you can say I received your patient B but maybe the fetal heart was not there so the midwife takes back that very book and she is also a senior midwife*. *So she doesn’t refer any patient out without that book because she wants us to write in that book the condition under which we received that patient*.

***PNFP*_*Hosp*_*001*_*IC*_*Mat***

The networks also consisted of unlikely community-level maternal health service provision actors for which the health system had to engage such as traditional birth attendants. Health workers embedded in these networks and familiar with this context would fast-track referrals from TBA and homes because they would have experienced multiple delays along the way to the facility. Although patients continued to be shared with this TBA, engaging her on how to improve timely referral has not been successful hence continued delays leading to the occurrence of as reflected in the quotation below;

*It is hard to engage her because one time we wanted to have a dialogue with her but they told me it’s a very hard thing she will not listen and a lot of people have been there so for her specifically we have not gone*.

***PNFP*_*Hosp*_*001*_*MO***

### Among non-state actors

Non-state actors were reported as active players and part of the local social network of maternal health services providers that supported the implementation of interventions to address stillbirth. NGOs, projects, and Professional Associations were some of the key players in the study area. Specifically, they would identify skills gaps among health workers and the health systems in general from which they intervened by providing the necessary support to address identified bottlenecks such as delayed access to maternal health services on the demand side and quality service on the supply side. Whereas some supported addressing demand-side barriers as reflected in the quote;

“*And we have a partner that [has] helped us a lot* … *from the USA*, *we have now designed an intervention of giving out incentives to the mothers when they come for the first ANC [to promote high completion rates]*

***(PNFP*_*Hosp*_*001*_*MO)***.

Others were involved in supply-side bottlenecks such as health worker training, management strengthening through support supervision, and infrastructural support among others.

*we have had representatives from Save the children and HBB Plus*. *They usually come and review with us [maternal and perinatal death reviews] and if there are any updates or new practices in the management of these babies perinatally then they share with us that information*. *Specifically*, *for stillbirths we have had Save the Children and Save the Mothers by the Association of Obstetricians and Gynaecologists of Uganda (AOGU)*. *I think those are some of the partners that we have worked with*.

***PNFP*_*Hosp*_*002*_*MO***

## Discussion

Our results suggest that health workers’ social networks influence the adoption of strategies to address the stillbirth burden at a subnational level health system in Uganda. We found evidence that health workers implementing maternal and child health policies at the subnational level worked within a social system that constituted an interwoven network. As such, through their supervision interactions and workplace relationships, these networks emerged among peers. These to a great extent influenced how health workers translated policies to address the stillbirth burden. Workplace networks had the greatest influence which was perhaps reflective of the importance of implementation context in directing policy outcomes. From the different levels of service delivery, the influence was more of linkage to other services not available at the point of care which may be reflective of limited resource context. It also reflected the hierarchical supervision relationships which were interwoven in the routine delivery of maternal health services at the subnational level. Among the private-for-profit providers, the influence was in form of caution and advice to stick to strict policy implementation expectations which may perhaps be reflective of the levels of trust in the ability of these actors to comply with policy provisions. Several studies in this area have established that these relationships exert varying amounts of influence on health workers’ practices and the eventual translation of policies into service delivery [6,13,14].

### Workplace social networks

Health workers spend more time with workplace-related social network members compared to any other networks. Our study revealed that members from the health workers’ places of work were the most dominant in influencing their practices which were done both through formal and informal mechanisms which included; coaching which embraced “learning by doing”, expert consultations from senior colleagues, occasional reminders about guidelines enforcement and peer learning through organized sessions. Other mechanisms of influence involved the implementation of quality assurance measures, skills-building to address identified gaps, support supervision, and the different interfaces through which staff feedback meetings. our results compare well with findings from elsewhere which established that contagion; the underlying social influence through personal interactions affected the adoption of new practices the more [10,11,23]. within the workplace setting, networks will inevitably emerge and these take on the professional outlook. It has long been established that professional social networks built based on professional consultation about patient care-related issues tend to influence practice in the direction of the mentor’s behaviours [13,24,25]. Direct persuasion as a form of social influence that is transmitted through direct communication among peers towards similar practices through cohesion [5] will tend to emerge in such settings. It is therefore key that while planning on the adoption of new practices regarding maternal health services, the influence of the workplace setting is not overlooked.

### Lower level to high-level service provision

Implementation context with performance expectations tends to influence the way actors behave. From this study, it emerged that while working in a health systems hierarchical setting at the subnational level, expectations from secondary level health facility workers tended to influence the way network members from primary level facility health workers supported interventions to address stillbirth risk. It was done through identification and recommendations for appropriate service providers, initiation of advance contact before effecting referrals, re-assessment of referred patients, and referral notes to identify inherent skills gaps. Networks emerging through a requirement for referral have been reported elsewhere [26,27]. As reported elsewhere [11] this is expected as individuals are driven by the normative desire to conform with socially similar peers through adaptation of behaviours to meet the peer expectations. In particular, the ego will adopt practices similar to those of peers (alters). It is triggered by self-reflection and evaluation of an individual’s standing about the subjective norm within a social context [1,28].

### Private and public health provider’s relationships

Our study also found that peer comparison between the private and public health service providers influences health workers’ adoption of strategies to address stillbirth. The expectation that private providers should try as much to emulate implementation practices of public health facilities elicited influence mechanisms which included; caution on service provision capacity according to the level of operation, advice, and mentorship on the appropriate use of drugs to prevent misuse of oxytocin during labor, support to data capture, adoption of proven evidence-based practices like the use of partograph for labor monitoring as well as looping lower-level private maternity service providers into the subnational referral system. Peer comparison is reported to trigger judgment based on structural equivalence as reference points to emulate those they regard as their peers even when they do not often interact directly [11,29]. Within the study setting, this led to efforts to ensure equal expectations from private providers as they implemented interventions to address stillbirth risks. The overall effects of peer comparison are reported to be determined by how the socially equivalent individuals relate to each other. From our study setting, although the private and public health facilities operate on different business models, they view themselves as providing complementary health services when it comes to interventions to address the stillbirth burden and maternal health in general. It is therefore key that once such interventions are rolled out equal attention and support being given to both the public and private maternal health service providers.

### From health facilities to district health managers interlinkages

The relationships among health workers and district health managers were also found to influence the way they adopted strategies to address stillbirth risk at the subnational level. This is expected as policy translation guidance is transmitted through district health managers to the respective health facilities. However, beyond this, some mechanisms can provide explanatory context to the observed behaviours. Within the subnational health system setting, many of the actors establish relationships with each other in a socially networked arrangement. Network members often fall back to these whenever specific tasks ought to be accomplished. From our results, it emerged that the mechanism to influence implementation included; engagement with particular staff to change practice behaviours, reminders to implement specific policy aspects such as the conduct of perinatal death reviews (PDR), expression of displeasure about high numbers of stillbirths which triggered action as well as collaboratively working together to address identified implementation bottlenecks. Similar findings were reported elsewhere reflecting physicians’ adoption of new practices being influenced by how their network members had adopted the same [6,30]. Where such guidance is from a shared source, practices are likely to reflect recommendations from the originator of the same [5] underscoring the influence of the social system on individual health worker practices. This finding also emphasizes the important role of the structure of the social networks in influencing the individual health workers’ adoption of practices [7,31]. Similarly, results have been reported about the influence of common contacts [1] and mutual friends [13,32] on other network members who in the long run may develop similar or comparable practices [14,33].

### Limitations

Our study had some limitations; first, the purposive sampling may have left out perspectives of other key actors, and therefore the results reported here may have represented a one-sided story more so from health workers directly involved in the implementation of MCH. This was addressed by the triangulation of results from the different data sources. Secondly, the study relied on information provided by the respondents, and in the context where relational exchanges among network members during the implementation of maternal and child health programs are not documented, views may be subjected to a recall bias. A key strength of this study is that the analysis and discussion draw on perspectives from multiple respondents at different levels of subnational health systems which enabled triangulation from more than one source and ensured the reliability of the information collected.

## Conclusions

Social network relationships among peers within a local subnational health system influenced the implementation and adoption of strategies to address the stillbirth burden through different mechanisms. This reflects the potential of social influence over the direction and success of policy implementation at the subnational level. Opportunities to strengthen these exposures through communication exist and are recommended which will go a long way in providing insights into underlying factors for policy adoption at the subnational level which ought to be harnessed for optimal policy outcomes.

## Supporting information

S1 Checklist COREQ checklist, description of data: Contains all key elements highlighted in the COREQ checklist, particular domains, their item numbers, the guide questions each responds to, and the page number within the manuscript on which such information is reported]. (DOCX)
